# United States response to the COVID-19 pandemic, January–November 2020

**DOI:** 10.1017/S1744133121000116

**Published:** 2021-03-05

**Authors:** Mathew Alexander, Lynn Unruh, Andriy Koval, William Belanger

**Affiliations:** 1 School of Medicine, Virginia Commonwealth University, Richmond, VA, USA; 2 Department of Health Management and Informatics, University of Central Florida, Orange County, FL, USA; 3 College of Pharmacy & Health Sciences, Campbell University, Buies Creek, NC, USA

**Keywords:** COVID-19 timeline, federal government response, trimodal peak

## Abstract

As of November 2020, the United States leads the world in confirmed coronavirus disease 2019 (COVID-19) cases and deaths. Over the past 10 months, the United States has experienced three peaks in new cases, with the most recent spike in November setting new records. Inaction and the lack of a scientifically informed, unified response have contributed to the sustained spread of COVID-19 in the United States. This paper describes major events and findings from the domestic response to COVID-19 from January to November 2020, including on preventing transmission, COVID-19 testing and contact tracing, ensuring sufficient physical infrastructure and healthcare workforce, paying for services, and governance. We further reflect on the public health response to-date and analyse the link between key policy decisions (e.g. closing, reopening) and COVID-19 cases in three states that are representative of the broader regions that have experienced spikes in cases. Finally, as we approach the winter months and undergo a change in national leadership, we highlight some considerations for the ongoing COVID-19 response and the broader United States healthcare system. These findings describe why the United States has failed to contain COVID-19 effectively to-date and can serve as a reference in the continued response to COVID-19 and future pandemics.

## 1. Introduction

Ten months have passed since the first case of coronavirus disease 2019 (COVID-19) in the United States (US), yet the outlook remains bleak. In late March, the US eclipsed China for the most confirmed cases in the world. The US now continues to lead the world in both COVID-19 cases and mortality. As of 25 November, there were nearly 13 million confirmed cases and greater than 262,000 deaths due to COVID-19 in the US (Dong *et al*., 2020).

The inadequate response to-date has been surprising. Global evaluations of pandemic preparedness prior to the COVID-19 pandemic had rated the US as one of the most well-prepared countries (Crosby *et al*., 2020).

The US also took early, albeit limited actions to combat COVID-19. On 7 January, the US created an Incident Management System to track and coordinate its public health response. Soon thereafter, the Centers for Disease Control (CDC), the nation's leading public health agency, held its first public briefing on COVID-19.

The first case of COVID-19, a recent traveller from Wuhan, China, was confirmed on 20 January in Washington state, 20 days after China had alerted the World Health Organization (WHO) of the new outbreak. The White House established a ‘Coronavirus Task Force’ and the US Department of Health and Human Services (HHS) declared a public health emergency. During January and February, the US began screening passengers at certain airports and asking travellers returning from high-risk areas to self-isolate.

**Figure 1.**
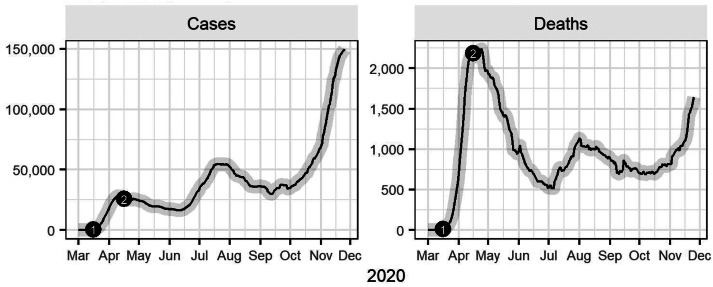
Confirmed cases and deaths from COVID-19 in the United States. (1) - Voluntary national shutdown instituted, limiting mass gatherings to 10 or less people, advising against discretionary travel, and recommending closure of schools, restaurants, gyms, and other indoor or outdoor venues. Extended through April 30. (2) - The White House released guidelines for state governors and local authorities to reopen the country. Source: JHU CSSE COVID-19 Data (https://github.com/CSSEGISandData/COVID-19)

Despite these steps, the US fell behind in its fight against COVID-19 and has failed to recover. As shown in Figure 1, the US has experienced a ‘trimodal wave’ in COVID-19 cases, with each peak higher than the previous one. The US set a new record of new cases daily in November, with a seven-day rolling average of almost 150,000 as of 25 November. As of the same date, more than 1600 Americans die daily from COVID-19, though the mortality rate has improved since the onset of the pandemic (Dong *et al*., 2020).

The pandemic has also affected racial and ethnic minorities and the poor disproportionally (Oberlander, 2020; Pollack, 2020), with higher rates of infections, hospitalisations and deaths among Blacks and Hispanics (CDC, 2020), and a greater number of hospitalisations among lower-income individuals (Munoz-Price, 2020). Poorer social determinants of health, such as education, employment, and housing, along with structural racism are important contributors to increased vulnerability to COVID-19 infection and poorer outcomes among minorities and the poor (Egede and Walker, 2020).

Given the pre-pandemic high ratings on pandemic preparedness and relative head start, why hasn't the US has been able to contain COVID-19 effectively? This paper describes key events and findings on the domestic response to COVID-19 from January to November 2020, reflects on the response to-date, and presents short- and long-term considerations for the US moving forward.

## 2. Overview of key events and findings

In this section, we review the US response using a template designed by the COVID-19 Health System Response Monitor (HSRM), a joint undertaking of the WHO Regional Office for Europe, the European Commission, and the European Observatory on Health Systems and Policies. The template, modified slightly, divides responses to COVID-19 into the following: (1) preventing transmission; (2) COVID-19 testing and contact tracing; (3) ensuring sufficient physical infrastructure and healthcare workforce; (4) paying for services; and (5) governance. Unless otherwise noted, citations are included in the US report on the HSRM website at https://www.covid19healthsystem.org/mainpage.aspx. Additional citations not in the HSRM report are noted in the text and referenced at the end of the article.

### 2.1 Preventing transmission

Federal and individual state governments have recommended or mandated key public health measures to contain the spread of COVID-19. In January and February, the federal government issued guidance on hand washing and respiratory etiquette. Physical distancing was advised for those who had tested positive, been in contact with a confirmed positive case, or recently visited a high-risk area. In late February, the federal government advised against close contact with those who were sick. In March, the CDC encouraged all high-risk individuals, defined as those over 60 or with underlying chronic health conditions, to avoid face-to-face contact.

On 16 March, President Donald Trump implemented a 15-day voluntary national lockdown (Figure 1). The guidelines limited mass gatherings to 10 people, advised against discretionary travel, and recommended closure of schools, dine-in restaurants, bars, and other public places. The CDC also advised all Americans to wear cloth face coverings in public areas such as grocery stores. The lockdown was subsequently extended to 30 April.

However, given the voluntary nature of the guidelines, there was no unified, enforced response across the country. Some states and localities took stronger measures. Twenty-nine states issued stay-at-home (SAH) orders by the end of March. Thirty states closed all non-essential businesses in March, 39 prohibited all gatherings or gatherings with more than 10 people, 44 closed restaurants and bars for dine-in seating, and 47 mandated school closures. Forty-two states had a mandatory SAH order in place at some point during the national lockdown.

In mid-April, the White House published a national reopening plan providing recommendations and reopening criteria to guide states and localities, again without mandates (Figure 1). The reopening criteria included a downward trajectory of cases, sufficient hospital capacity, and robust testing infrastructure. The three-phase plan called for gradual loosening of public health measures like limits on public gatherings and restrictions on non-essential businesses.

Several states, however, reopened prior to the end of the national lockdown. As of 24 April, eight states had started removing physical distancing restrictions. Neighbouring states formed regional reopening coalitions, including in the East, West, and South. Several states in the southern coalition began reopening despite not meeting key criteria like testing capacity.

**Figure 2.**
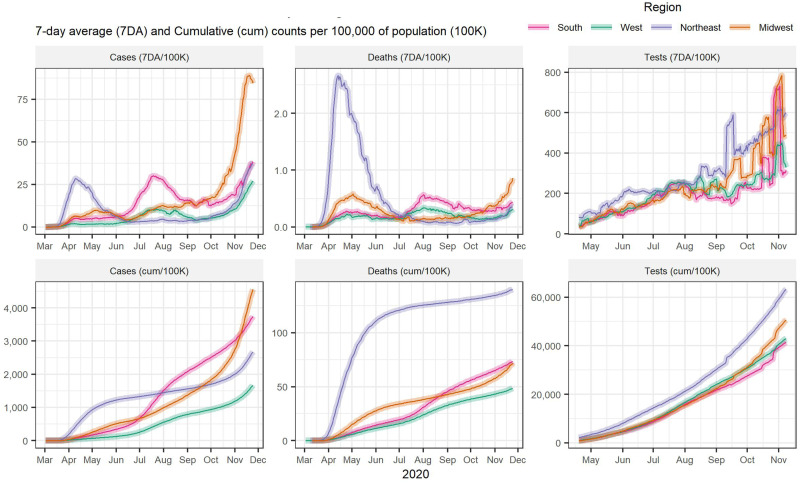
COVID-19 cases, deaths and tests by US region. Source: JHU CSSE COVID-19 Data (https://github.com/CSSEGISandData/COVID-19)

By June, all states had started reopening. A new surge in cases during the summer caused some to slow reopening. By early August, 17 states imposed new restrictions and seven paused reopening. As illustrated in Figure 2, this second peak in cases occurred primarily in the South (states that had reopened too early and/or too fast) as opposed to the initial spike in the Northeast.

As cases dipped during late summer and early fall, the US proceeded with increased reopening. For instance, some schools and universities reopened for either partial or complete in-person learning. But a new spike in cases (Figure 2), particularly in the Midwest, led some states to again reintroduce mitigation measures.

Mask-wearing, which has been politicised throughout the pandemic, has also been a key, but under-utilised, tool. Studies have demonstrated that jurisdictions with mask mandates have experienced decreased transmission compared to their mandate-less counterparts, including a comparison of counties in Kansas with differing masking policies (Van Dyke *et al*., 2020). Yet, as of November, several states still lacked mask mandates.

### 2.2 COVID-19 testing and contact tracing

In addition to broader public health measures, building testing and contact tracing infrastructure has been crucial to identifying and isolating COVID-19 positive individuals and limiting spread.

Initial testing development and capacity were hindered by defective tests and slow enlistment of private laboratories. The Food and Drug Administration (FDA) later authorised private and state and local public health laboratories to develop tests in February. The first commercial coronavirus test was approved on 13 March.

With public−private sector involvement and advent of new tests, testing capacity began increasing as seen in Figure 2. By late April, one to two million people were tested weekly on average, though this still fell short of recommendations from public health experts. As of 25 November, nearly two million tests were conducted daily.

Despite increased testing capacity, testing issues persisted. Early in the pandemic and during periods of increased demand, testing has been prioritised for symptomatic individuals, travellers returning from high-risk areas, and individuals in other high-risk groups like healthcare workers. Test reporting delays, coupled with an earlier lack of rapid, point-of-care testing, hindered contact tracing and containment efforts. Testing kit and supply shortages, further limited widespread availability of testing. Testing sites have also been disproportionately distributed, including being sparse in communities with racial and ethnic minorities and rural areas. Finally, the lack of a federal testing strategy has largely left states to handle purchases and distribution. Some states have created multi-state purchasing compacts to overcome this roadblock.

Contact tracing has faced similar hurdles. Public health experts initially called for more than 100,000 contact tracers, yet the US only reached 50,000 in October (Simmons-Duffin, 2020). States have additionally released digital tracing apps to supplement human contact tracing efforts. But privacy concerns have limited their uptake.

### 2.3 Ensuring sufficient physical infrastructure and healthcare workforce

Healthcare institutions in the US stock inventory very leanly (Rice *et al*., 2020), so as COVID-19 infections accelerated throughout February and March 2020, the existing supply of physical resources quickly fell behind demands. Shortages of ICU beds, ventilators, testing equipment, personal protective equipment (PPE) and other medical equipment and supplies ensued.

Procuring medical equipment and supplies and expanding bed capacity for COVID-19 cases proceeded slowly in February. The explosive growth in COVID-19 cases in March−April created a strain on the infrastructure.

Shortages were addressed through a mix of national, regional/local and private initiatives, and by reorganising healthcare delivery. Initially, the Federal Emergency Management Agency (FEMA) distributed its Strategic National Stockpile to hard-hit areas, but shortages remained in some areas. Supplies quickly ran out. The Coronavirus Aid, Relief, and Economic Security (CARES) Act provided funding for producing and purchasing ventilators and masks. The Defense Production Act, which gives federal agencies the authority to require companies to prioritise government contracts for medical supplies, funded production of ventilators and N95 masks. Other than these federal initiatives, states, local areas, and individual hospitals were responsible for obtaining needed equipment and supplies.

In mid-March, routine healthcare services, such as annual check-ups and elective procedures, were postponed, and excess capacity was utilised for COVID-19 care. Medical−surgical units were converted to ICU beds and healthcare workers were moved to areas of greatest need. These services were restarted in the summer. Telehealth services were built up through waivers granting states relaxation of regulations of virtual visits, and reducing out-of-pocket expenses.

These efforts to reorganise services and produce needed equipment, combined with a fall in hospital resource use in late April, alleviated the shortage of hospital beds and ventilators somewhat by May. However, as several states reopened in May and June, and cases surged, having sufficient ICU beds once again became an issue in July. In Arizona and Florida, ICU beds were at capacity by early July. With the fall surge, shortages of hospital beds has reappeared, particularly in the Midwest and South. PPE shortages continued throughout the pandemic (see Box 1).

Box 1. PPE shortages

**PPE shortages:**

- In late March, nearly a third of healthcare facilities were almost out of face masks, 13% were out of face shields, and about 25% were completely or nearly out of gowns (Kamerow, 2020).
- A national survey of nurses from 15 April to 10 May found that 87% of nurses had to reuse PPE designed to be single-use, and 72% had worked with exposed skin or clothing while treating COVID-19 patients (National Nurses United, 2020a, 2020b).
- Nursing homes have had severe PPE shortages (McGarry *et al*., 2020).
- As of mid-September, hospitals, nursing homes, and doctor's offices still had to decontaminate disposable masks and gloves for reuse and to hunt for critically needed equipment through unconventional markets (Finkenstadt *et al*., 2020).

The initial healthcare workforce supply going into the pandemic was on the low side. The US has fewer practicing physicians and fewer nursing staff working in hospital patient care compared to other high-income countries (Rice *et al*., 2020; Kamal *et al*., 2020).

As the pandemic grew in spring 2020, shortages of health professionals developed in pandemic epicentres, particularly in critical care and emergency rooms. Shortages eased in acute care settings in May as the number of new cases began flattening. Workforce requirements then shifted to public health workers who could carry out testing, contact tracing, and quarantining those exposed.

Acute care staffing shortages appeared again in the summer and fall with the surges in the Southern and Midwestern states, respectively. As of 19 November, hospitals in 25 states were critically short of clinicians and other staff (Goldhill, 2020). More than 20 per cent of nursing homes have also had staff shortages since May (McGarry *et al*., 2020).

Healthcare worker shortages were exacerbated due to continued PPE shortages and testing difficulties. Insufficient PPE and testing has contributed to 1.3 times higher infection rates among healthcare workers (NNU, September 2020). As of September 2020, over 1700 healthcare workers have died from COVID.

The situation has become dire. Doctors and nurses are experiencing mental exhaustion, post-traumatic stress disorder, and burnout (Abelson, 2020). A July survey reported that around 8% of doctors had closed their offices and another 4% planned to within the next year (Abelson, 2020). Nurse turnover rates are high and nurses are leaving the profession (Abelson, 2020). Southern California, Houston and Illinois nurses went on strike in July, Northern California nurses struck in October, and Pennsylvania nurses struck in November over inadequate PPE, unsafe working conditions and understaffing.

### 2.4 Paying for services

Additional health financing has helped mitigate excess demands on the health care system and bolster the public health response.

In March and April, Congress passed four major bills addressing COVID-19: (1) the Coronavirus Preparedness and Response Supplemental Appropriations Act, an USD $8.3 billion bill that provided funding to states and localities for COVID-19 preparedness and response; (2) the Families First Coronavirus Response Act (FFCRA), which addressed insurance coverage of coronavirus testing, paid sick leave, nutrition assistance, and unemployment benefits; (3) the CARES Act, which included more than USD $150 billion for hospitals, research, treatment, and stockpiling equipment and an additional USD $150 billion for state and local governments to invest in capabilities like testing and contact tracing infrastructure; and (4) the Paycheck Protection Program and Health Care Enhancement Act, which allotted USD $75 billion to hospitals and $25 billion to increase testing capacity.

Key actions have also supported individuals. The FFCRA waived co-payments for COVID-19 testing for Americans with all types of insurance, including private, Medicare, and Medicaid. The federal government expanded telehealth benefits for Medicare beneficiaries. Other insurers similarly waived co-payments and deductibles for inpatient admissions and telehealth services. Some insurers further waived cost-sharing requirements for all COVID-19-related services, removed prior authorisation, and eliminated out-of-network requirements.

A patchwork of policies has supported coverage for the uninsured, including 5.4 million Americans who lost employer-sponsored health insurance between March and May. For services aside from acute hospital care, the uninsured have had a few options to obtain insurance to avoid paying out-of-pocket: the Consolidated Omnibus Budget Reconciliation Act, which allows individuals to keep their employer coverage after losing their job, Medicaid/CHIP, or a subsidised Affordable Care Act plan. Acute hospital care would most likely be covered through charity care, though the CARES Act provided hospitals with relief funding to cover such uncompensated care.

Despite these actions, health financing issues have arisen. Congress did not pass additional stimulus funding between April and November, placing small businesses and individuals at-risk of financial ruin. CARES Act funding disproportionately favoured hospitals with higher shares of private insurance, negatively impacting Medicaid providers and safety-net hospitals (Schwartz and Damico, 2020). While diagnostic testing is covered, some insurers do not cover asymptomatic or surveillance testing. Moreover, surprise medical billing practices for out-of-network labs and providers have continued despite earlier HHS attempts to prevent such practices.

### 2.5 Governance

The COVID-19 outbreak was declared a public health emergency on 31 January and a national emergency on 13 March. To advise and communicate response efforts at the federal level, the President formed the Coronavirus Task Force on 29 January. Members included the Vice-President (chair), scientists such as Drs. Fauci and Birx, the heads of HHS and CDC, and other officials. However, increasingly, the Trump Administration openly disagreed with the scientists on the committee, and by late April, the Task Force met with the President infrequently and made few public briefings. Although the Task Force still exists as of November, it has very little public presence.

The Task Force made FEMA the lead response organisation on 19 March, weeks after the pandemic had started. FEMA began providing supply assistance to the states, local governments, and private non-profit organisations in late March, but distribution was uneven, lacked transparency and has not been sustained. Starting mid-March, the Defense Production Act was employed a few times, but not in a long-term or widespread manner (e.g. to relieve ongoing PPE or testing supply shortages).

The CDC has coordinated surveillance, testing, and international reporting, and published a number of guidelines on pandemic containment/mitigation and safe reopening (e.g. reopening schools). Despite these actions, the CDC has had little public presence and its guidance is increasingly vetted by the White House.

As the Coronavirus Task Force and CDC were marginalised and the Trump Administration handed resource allocation and containment/mitigation decisions to the states, leadership devolved to state and local governments. The National Governors Association coordinated state governments. Little federal guidance existed on how to respond to the resurgence of cases occurring in many states. Affected states made their own decisions, some slowing or reversing reopening, others deferring decisions to local authorities (counties or cities), yet others continuing reopening and making it difficult or impossible for local authorities to do otherwise.

State-wide orders for physical distancing and mask-wearing tended to fall along political lines as Republican governors were less likely to issue mandates than Democratic governors (Adolph *et al*., 2020). For instance, despite a large surge in cases in Florida in June and July, Florida's Republican governor did not issue a mask mandate, but several local authorities did. Georgia's Republican governor barred local mask mandates, but local authorities defied the ban. Facing big surges in infections and full hospitals in the Midwest in November, some Republican governors and/or legislatures, are beginning to reverse their opposition to mandates.

Official public briefings about the pandemic by the Trump Administration declined over time, but when they occurred, the message was likely to contradict and marginalise public health officials, scientists, and guidelines. The administration often downplayed the seriousness of the pandemic, placing a positive spin on the situation in the US. President Trump personally set examples that contradicted public health guidance (e.g. held indoor rallies where masks were discouraged, refused to wear a mask). Such actions and politicisation of important public health tools by leadership led to a lack of public compliance with mask-wearing and physical distancing (New England Journal of Medicine, 2020).

In mid-July, the CDC was relieved of its duty of collecting data on COVID-19 hospitalisations. Instead, the information was sent to HHS, an agency under strong administration influence, and funnelled through Teletracking, a small private firm that was controversially awarded the contract. This decision has resulted in long delays and data errors, further delaying the ability of public health officials to determine clusters and patterns and predict needed resources (Huang and Simmons-Duffin, 2020).

The Trump Administration also interfered with pandemic guidelines. In May, the CDC's initial detailed reopening guidelines were delayed while a shorter version was released. A more detailed version was later published on the CDC website incorporating changes. On 1 July, the CDC developed school reopening guidelines that recommended that schools collaborate with state and local health officials to determine how classes should be taught in the fall and outlined the risks of different teaching configurations. President Trump felt that the guidance was ‘too tough and expensive,’ and directed the CDC to create different guidelines (White House, 2020). In September, administration officials in HHS published testing guidelines on the CDC website (bypassing the CDC's scientific review process), stating that people who have been exposed to COVID-19 do not need testing if they are asymptomatic. This was quickly corrected by CDC scientists. In October, the administration installed two officials without public health backgrounds at the CDC to monitor CDC officials and try to control the information the CDC releases (Dearen *et al*., 2020). Similar political interference has been noted in other government agencies, including the FDA, where earlier political pressure to expedite approval of a potential COVID-19 vaccine contributed to ongoing concerns about safety and efficacy.

## 3. Reflections on the US response

The findings above highlight several key actions (or inactions) that may have been crucial to the US COVID-19 experience. These actions may have affected containment, and therefore the numbers of cases and deaths from the virus. As our findings are descriptive in nature, the lessons we draw from them are tentative. Below, we summarise these actions and discuss how they may have affected the progress of the pandemic. Then, we present a figure that shows the interface of some actions with trends in cases in three states representative of the three COVID-19 surges in the US.

1. Initial containment measures (shutting borders, locking down) were delayed and were not mandated nationally.
  COVID-19 cases first appeared in the US on 20 January, but lockdowns did not occur until mid-March, and these were not nationally mandated. By late March, COVID-19 cases and deaths were surging. Earlier mandated national lockdowns might have resulted in less community spread in the initial surge.
2. Testing got off to a slow start, never reached consistent sufficient capacity, and testing times remained too long, thereby impeding contact tracing.
  A slow start to testing likely contributed to the surge in cases in March. Testing and contact tracing capacity have increased significantly, but some areas remain below capacity, and test reporting delays impede timely tracing. Areas that experience testing access problems and delays in reporting may experience greater surges. The lack of a national testing and tracing strategy is a major contributor to these issues, and raises concerns about potential vaccine administration.
3. Initial physical and human resources were insufficient for a pandemic response and there were challenges building them up.
  The practice of ‘just in time’ in US healthcare and endemic shortages of healthcare professionals, produced shortages of needed resources that were difficult to overcome. Future pandemic preparation should be mindful of the precariousness of these lean practices and should build up a more secure starting place and greater stockpiles and methods for scaling up in the event of a pandemic.
4. National leadership abdicated, and leadership fell to the states.
  By May, leadership devolved to state and local governments. States and localities developed their own testing and containment/mitigation measures and resource allocation. States with lower testing capacity and less adherence to public health guidelines may have created conditions for greater viral spread (e.g., see #5).
5. Some states may have reopened too early and quickly.
  Most states reopened in May, some as early as the end of April. Not all followed CDC guidelines regarding readiness for and pace of reopening. A second wave in Southern states occurred in June–August, and a third wave in Midwestern states started in September. The summer wave may have been due to the states not following CDC suggestions for reopening safely and lack of mandates. The fall wave may have been due to cold weather moving people inside more, school reopenings (particularly universities), and inadequate containment/mitigation measures. Based on finding other high-income countries reopening too early, Han *et al*. (2020) suggest gradual reopening with adequate monitoring.
6. Noncompliance with mandates/guidelines may also have contributed to all three surges.
  In contrast to several Asian countries where compliance with containment measures has been high (Han *et al*., 2020), in the US, some have not complied with measures such as wearing masks, avoiding crowded restaurants and bars, and not gathering in large confined groups. Noncompliance may be due to the conflicting messages between the Trump Administration and public health experts, lack of experience with pandemic restrictions (Han *et al*., 2020), ‘pandemic fatigue’ (WHO, 2020), and politicisation of adhering to the containment/mitigation measures, where Trump supporters are less likely to adhere to containment measures than Democratic supporters (Gollwitzer *et al*., 2020).
7. Lack of entitlements to care may have affected access to testing, treatment and outcomes.
  Coronavirus coverage for the uninsured has not been easy to obtain. This may lead to delays in testing and treatment (Khatana and Groeneveld, 2020). This may be particularly damaging for racial and ethnic minorities and socioeconomically disadvantaged groups that have been disproportionately impacted by COVID-19 (Azar *et al*., 2020).
8. The Trump Administration marginalised public health and scientific leaders, and contradicted and interfered with scientific guidelines and advice.
  The Trump Administration's disregard for and active interference in science and public health led to inconsistent messaging and misinformation, which may have contributed to lack of proper containment/mitigation measures by some states and noncompliance with recommended measures by some of the populace (New England Journal of Medicine, 2020). This could have contributed to increased community spread than if the administration had cooperated with public health leaders.
9. The healthcare workforce was not protected.
  Healthcare personnel and PPE shortages, and inadequate testing of healthcare workers may have led to further shortages of healthcare workers (Nguyen *et al*., 2020) and to their physical and mental distress. These issues need to be addressed to lower the stress of healthcare workers and retain them in the workforce.
10. Containment measures were politicised, and behaviour had a partisan basis.
  State variation in testing capacity, timing of and preparedness for reopening, and containment/mitigation measures appear to have a partisan basis (Adolph *et al*., 2020; Gollwitzer *et al*., 2020). Consistent with messaging from President Trump, many Republican-controlled states issued SAH orders later, refused to mandate mask-wearing, and reopened their economies sooner. Politicisation of mask-wearing and other containment/mitigation measures has contributed to the lack of collective action and possibly to inadequate containment. The issue of partisanship is difficult to deal with, as political views are not easily swayed by reason. A change in national leadership, along with positive messaging on containment measures, may reduce some opposition to these measures (New England Journal of Medicine, 2020). Public health, community, and business messaging on the reality of the pandemic and the importance of the containment/mitigation measures may also help (Sherling and Bell, 2020).

**Figure 3.**
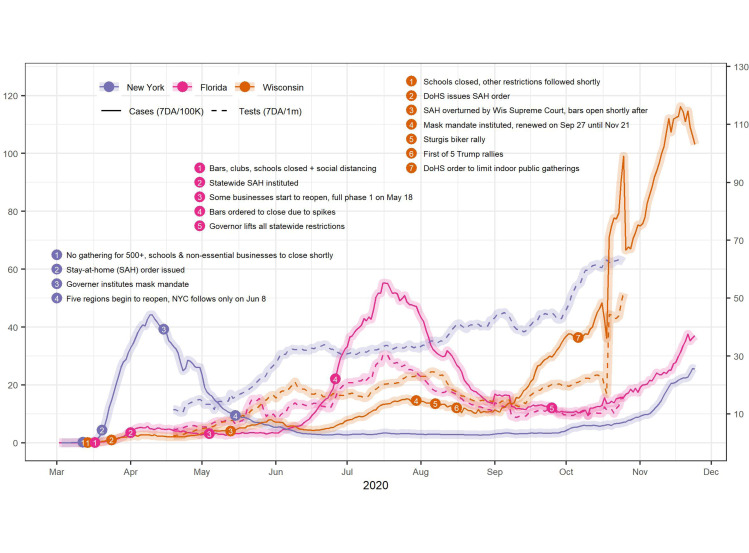
COVID-19 cases and tests in three US states. Source: JHU CSSE COVID-19 Data (https://github.com/CSSEGISandData/COVID-19)

New York, Florida and Wisconsin are three states that are representative of the three surges in the US. New York experienced a severe surge in the spring, Florida in the summer, and Wisconsin in the fall. Figure 3 presents the population-adjusted 7-day rolling average of COVID-19 cases in each of these states. Along the trend lines for these states are bullets that indicate notable events and actions. Dotted trend lines show the population-adjusted 7-day rolling average of tests in those states.

The New York trend line indicates that it led in surges in the spring while other states had only small increases in cases. New York was one of the first states to have a serious surge due to its metropolitan nature and international connections. The SAH orders and mask mandates appear to have helped bring the surge under control within a month, along with other measures not noted in the figure. New York reopened mid-May, but maintained certain containment measures such as mask mandates. This appears to have helped keep cases low until October. Colder weather may be driving the fall uptick.

In Florida, as cases began to rise in March, bars were closed on 17 March and a state-wide SAH order was initiated on 1 April. The state experienced a small increase in cases in April−May. Florida reopened in early May without sufficient testing and tracing and without a mask mandate, although phased physical distancing rules were instituted. A surge in cases began in June. Bars were closed again on 26 June (no other change in reopening) and the surge was brought down, but never to baseline. All state-wide restrictions were lifted 25 September and cases were increasing again in November.

In Wisconsin, containment measures began on 14 March and SAH orders followed on 24 March. The Wisconsin Supreme Court overturned the SAH order on 13 May. Shortly thereafter, cases began to rise. A mask mandate issued on 30 July is associated with the figure with a subsequent drop in cases. However, Wisconsin residents attended a motorcycle rally in Sturgis, South Dakota from 7 to 16 August. The rally has been cited as a super-spreader event for nearby states. Large Trump rallies, where many people were maskless, occurred in October and November. Since 1 September, Wisconsin has seen cases skyrocket.

Figure 3 also shows that New York performs much more testing than the other states, even Wisconsin, which increased testing to a high level in the fall. Wisconsin's increased testing may be in response to the high number of cases. In contrast, New York has high testing but low positive numbers, possibly indicating reverse causation – cases are low due in part to extensive testing and tracing.

These figures provide some evidence for the efficacy of several containment measures: SAH orders, physical distancing, wearing masks, and high testing capacity. State-wide mask mandates appear to be more effective than letting localities decide.

## 4. Looking ahead

The near-term future offers little hope for a quick turnaround. The current wave of increasing cases will inevitably be followed by increased mortality. Holiday travels and gatherings during Thanksgiving and Christmas may add to surging cases nationwide. A converging flu season will further complicate efforts to mitigate and trace spread.

Nonetheless, the fall has brought some promise. Vaccine development is making significant progress, with three candidates showing encouraging efficacy data in phase III trials and moving towards emergency approval. As these vaccines and others in the pipeline edge closer to approval and mass distribution, prioritisation, accessibility, affordability, and supply chain management considerations will arise. Therapeutic development has similarly boded well. Treatments like dexamethasone and monoclonal antibodies have received emergency approval, though widespread access remains limited.

In November, President-Elect Joe Biden announced a 13-member COVID-19 Task Force composed of physicians, scientists, and public health experts. These and other key administration appointments and the proposed Biden COVID-19 response plan suggest the potential for a more scientifically driven, federal government-led response.

The long-lasting impact of this crisis on systemic issues – health insurance coverage, health care costs, access to care, and social determinants of health – remains to be seen. Millions lost employer-sponsored health insurance due to job losses, illustrating the lack of universal coverage in the US. States with expanded Medicaid were better positioned to respond to COVID-19, such as leveraging Medicaid as a safety net for the uninsured, including those losing employer coverage. The pandemic may lead the charge for universal coverage, perhaps through expansion of public insurance.

Finally, COVID-19 has illustrated the importance of non-healthcare policies such as eviction moratoriums and paid sick leave in influencing health and healthy behaviours. Future investment in public health must include consideration of important social factors like housing, food, and transportation that impact health.
